# An Unusual Case of Sinusitis in a Flight Attendant

**DOI:** 10.7759/cureus.55639

**Published:** 2024-03-06

**Authors:** Nicholas Kramer, Scott Manthei

**Affiliations:** 1 Medicine, Touro University Nevada, Henderson, USA; 2 Otolaryngology, Nevada Ear and Sinus Institute, Las Vegas, USA

**Keywords:** air travel, dental amalgam, foreign body, sinus barotrauma, ear nose throat (ent), recurrent sinusitis

## Abstract

Foreign body sinusitis is a rare but important condition that should be taken into account when considering differential diagnoses. In this case report, we present a unique case of sinusitis caused by a foreign body originating from a dental procedure. Additionally, the complexity of the case was compounded by the patient's occupation as a flight attendant. A 49-year-old female flight attendant presented with a two-month history of facial pressure exacerbated by flying. A computed tomography (CT) of her paranasal sinuses confirmed the presence of a radiopaque foreign body near the left maxillary infundibulum, with minimal left ethmoid sinus mucosal thickening. Initially, she elected for non-operative management due to schedule conflicts. Upon follow-up over the next year, she complains of recurring severe facial swelling and congestion. A repeat CT scan shows that she has a dental amalgam that migrated from her left maxilla to the ethmoid infundibulum, lodged between her uncinate process and ethmoid bulla. The patient subsequently underwent foreign body removal. The patient recovered well, and a follow-up CT confirmed the complete removal of the foreign body. This case provides excellent insight into the mechanism of foreign body migration and sinusitis complicated by the unique circumstances of barotrauma associated with regular air travel.

## Introduction

Foreign body (FB) sinusitis is a rare but important differential diagnosis to consider among presentations of chronic sinusitis. A thorough medical history, including interdisciplinary information from fields such as dentistry, can be advantageous in identifying the etiology of FB sinusitis. Furthermore, it is vital to consider the occupation of patients with potential FB sinusitis as some career fields, such as marine or aviation work, can expose the patient to pressure changes and subsequent barotrauma. Barotrauma is physical tissue damage caused by an unrelieved pressure difference between surrounding gas or fluid and a canceled body cavity, such as the sinuses [1]. These previously stated factors may accelerate FB migration within the sinuses. This case report explores a patient who uniquely fits the previously stated factors. Informed consent was obtained from the patient.

## Case presentation

A 49-year-old female flight attendant presented with a two-month history of facial pressure exacerbated by flying. She describes feeling pain and pressure localized to the midface. She attributed these symptoms to a metal FB in her sinuses, which was incidentally discovered during dental imaging conducted previously. The patient had no prior history of sinusitis and denied any head trauma or nasal surgeries, although she did mention extensive dental work. Additionally, she had undergone bilateral retinal surgeries in the past.

A complete otorhinolaryngological examination, including rigid endoscopy, revealed hypertrophic turbinates and a mild left septal deviation. Neurologic and ophthalmologic examinations yielded normal results.

A computed tomography (CT) of her paranasal sinuses confirmed the presence of a radiopaque FB measured at 3 mm near the left maxillary infundibulum, with minimal left ethmoid sinus mucosal thickening, and the remaining sinus cavities were noted to be normal. For personal reasons, she requested non-operative management at this time.

**Figure 1:**
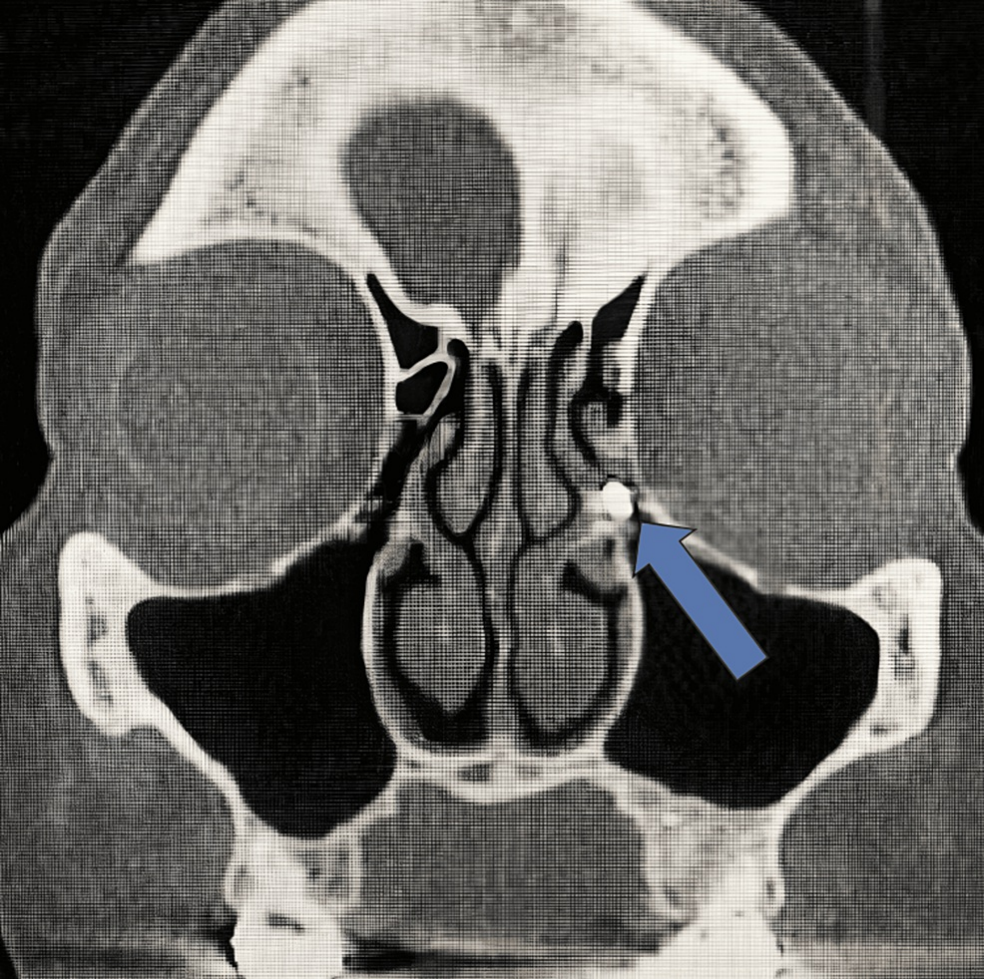
Metal foreign body in the ethmoid infundibulum

Over the next year, the patient complained of recurring severe facial swelling and congestion; antibiotics helped but never resolved her symptoms. After repeating a CT scan of the sinuses (Figure 1), it appears most likely that she has a dental amalgam that migrated from her left maxilla to the ethmoid infundibulum, lodged between her uncinate process and ethmoid bulla. The metal FB appears to erode into the ethmoid bulla and ethmoid air cell mucosal thickening. Plausibly, the migration of the FB and subsequent erosion caused was expedited by the barotrauma associated with frequent air travel as a flight attendant.

Given her previous history of ocular surgery, the patient expressed concern with the continued migration of the FB through the ethmoid sinus toward the orbit. With this history, coupled with the chronic pain compounded by regular barotrauma, the patient desired to proceed with endoscopic sinus surgery. The patient underwent general endotracheal anesthesia with left maxillary debridement, left anterior ethmoidectomy, and FB removal. A somewhat retracted left uncinate process was noted, but no significant polypoid degeneration or abnormalities were noted. The sphenoethmoid recess and nasopharynx were clear. The uncinate process and ethmoid bulla were taken down, and a calcified mass was removed from the left infundibulum between the maxillary and ethmoid sinus.

## Discussion

Migration of FBs into the maxillary sinus is becoming more prevalent with the increasing use of dental implants [2]. Sinusitis caused by FB is uncommon but important to consider among differential diagnoses. The frequency of sinusitis caused by FB is challenging to determine due to the event's rarity. However, among paranasal sinus FBs, most are iatrogenic (60%), while fewer occur from accidents (25%) [3]. Within iatrogenic etiologies, dental procedures are the leading cause.

The mechanism of sinusitis caused by FB remains undetermined. However, it has been hypothesized that metal FBs cause chronic chemical and physical irritation of the mucosa, leading to partial or complete ciliary dysfunction, resulting in a lack of mucus clearance and sinus congestion [4]. Mucociliary clearance is crucial to the normal function of the paranasal sinuses, and future research on the mechanism of FB sinusitis would be beneficial.

Mucociliary flow has been attributed as the mechanism of FB migration [5]. The natural flow begins at the maxillary floor and rhythmically beats mucus to the maxillary ostium and out into the infundibulum; this narrow space is where FB lodging is likely to occur [6]. Additionally, sinus tissue injury can be caused by sinus barotrauma. Sinus barotrauma injury is caused by rapid changes in intrasinusal air versus the surrounding atmosphere [7]. Sinus barotrauma is common in both air-flight passengers and scuba divers. The existing body of literature extensively discusses the mucosal injury resulting from FBs and barotrauma separately [8-11]. Nevertheless, no literature has been identified that particularly addresses the combined impact of these two processes. Considering the patient's presentation, it is rational to infer that the simultaneous occurrence of barotrauma and FB has accelerated the rate of tissue injury.

## Conclusions

The importance of assessing the complete clinical picture in the setting of FB sinusitis is highlighted by this case. This case provides a unique illustration of FB mucosal injury compounded by occupational barotrauma, which should prompt clinicians to consider the patient's occupational background when making clinical decisions for individuals with FB sinusitis. Furthermore, interdisciplinary history, particularly in dentistry, is essential in investigating the etiology of sinonasal FBs.
